# Supplementary material for: Novel Driver Strength Index highlights important cancer genes in TCGA PanCanAtlas patients
Source: PeerJ. 2022 Aug 11;10:e13860. doi: 10.7717/peerj.13860 (PMC9375969; doi:10.7717/peerj.13860)
Supplement: Supplemental Information 2 [file peerj-10-13860-s002.zip › COHORTS/genes plots/cohorts/2021_8_16_14_9_distribution_cohorts_females_KIRP.pdf]

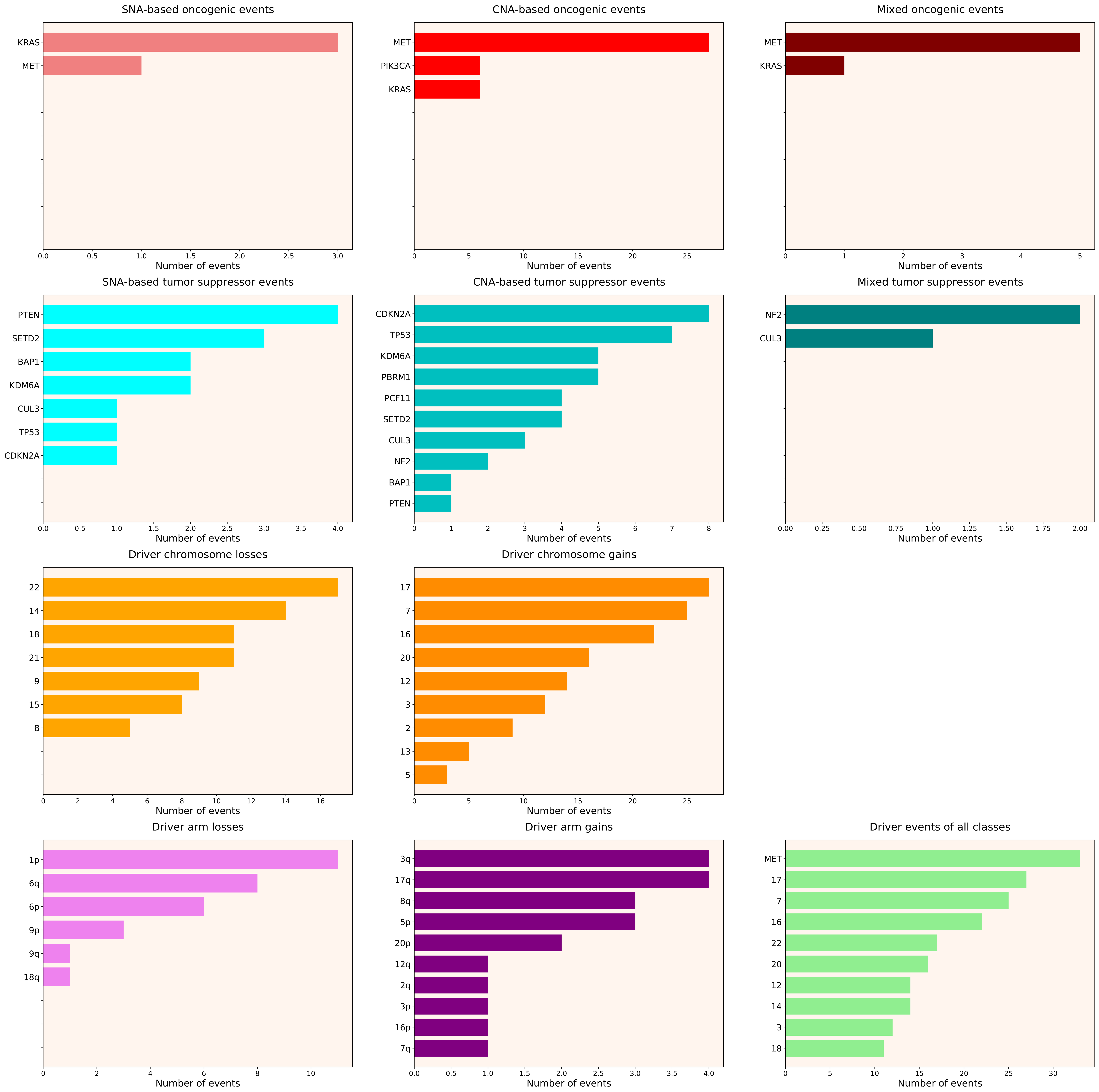

SNA-based oncogenic events
KRAS
MET
Number of events
CNA-based oncogenic events
MET
PIK3CA
KRAS
Number of events
Mixed oncogenic events
MET
KRAS
Number of events
SNA-based tumor suppressor events
PTEN
SETD2
BAP1
KDM6A
CUL3
TP53
CDKN2A
Number of events
CNA-based tumor suppressor events
CDKN2A
TP53
KDM6A
PBRM1
PCF11
SETD2
CUL3
NF2
BAP1
PTEN
Number of events
Mixed tumor suppressor events
NF2
CUL3
Number of events
Driver chromosome losses
22
14
18
21
9
15
8
Number of events
Driver chromosome gains
17
7
16
20
12
3
2
13
5
Number of events
Driver arm losses
1p
6q
6p
9p
9q
18q
Number of events
Driver arm gains
3q
17q
8q
5p
20p
12q
2q
3p
16p
7q
Number of events
Driver events of all classes
MET
17
7
16
22
20
12
14
3
18
Number of events
